# Fatal Congenital Heart Disease in a Postpartum Woman

**DOI:** 10.3390/diagnostics15151952

**Published:** 2025-08-04

**Authors:** Corina Cinezan, Camelia Bianca Rus, Mihaela Mirela Muresan, Ovidiu Laurean Pop

**Affiliations:** 1 Department of Medical Disciplines, Faculty of Medicine and Pharmacy, University of Oradea, 410073 Oradea, Romania; rus.cameliabianca@student.uoradea.ro; 2 Clinical County Emergency Hospital Bihor, 410169 Oradea, Romania; muresan.mihaelamirela@student.uoradea.ro (M.M.M.); popo@uoradea.ro (O.L.P.); 3 Doctoral School of Biological and Biomedical Sciences, University of Oradea, 410087 Oradea, Romania; 4 Department of Morphological Disciplines, Faculty of Medicine and Pharmacy, University of Oradea, 410073 Oradea, Romania

**Keywords:** atrioventricular defect, Eisenmenger syndrome, single atrioventricular valve, clubbed fingers, right-to-left shunt

## Abstract

The image represents the post-mortem heart of a 28-year-old female patient, diagnosed in childhood with complete common atrioventricular canal defect. At time of diagnosis, the family refused surgery, as did the patient during her adulthood. Despite being advised against pregnancy, she became pregnant. On presentation to hospital, she was cyanotic, with clubbed fingers, and hemodynamically unstable, in sinus rhythm, with Eisenmenger syndrome and respiratory failure partially responsive to oxygen. During pregnancy, owing to systemic vasodilatation, the right-to-left shunt is increased, with more severe cyanosis and low cardiac output. Echocardiography revealed the complete common atrioventricular canal defect, with a single atrioventricular valve with severe regurgitation, right ventricular hypertrophy, pulmonary artery dilatation, severe pulmonary hypertension and a hypoplastic left ventricle. The gestational age at delivery was 38 weeks. She gave birth to a healthy boy, with an Apgar score of 10. The vaginal delivery was chosen by an interdisciplinary team. The cesarean delivery and the anesthesia were considered too risky compared to vaginal delivery. Three days later, the patient died. The autopsy revealed hepatomegaly, a greatly hypertrophied right ventricle with a purplish clot ascending the dilated pulmonary arteries and a hypoplastic left ventricle with a narrowed chamber. A single valve was observed between the atria and ventricles, making all four heart chambers communicate, also insufficiently developed interventricular septum and its congenital absence in the cranial third. These morphological changes indicate the complete common atrioventricular canal defect, with right ventricular dominance, which is a rare and impressive malformation that requires mandatory treatment in early childhood in order for the condition to be solved.

**Figure 1:**
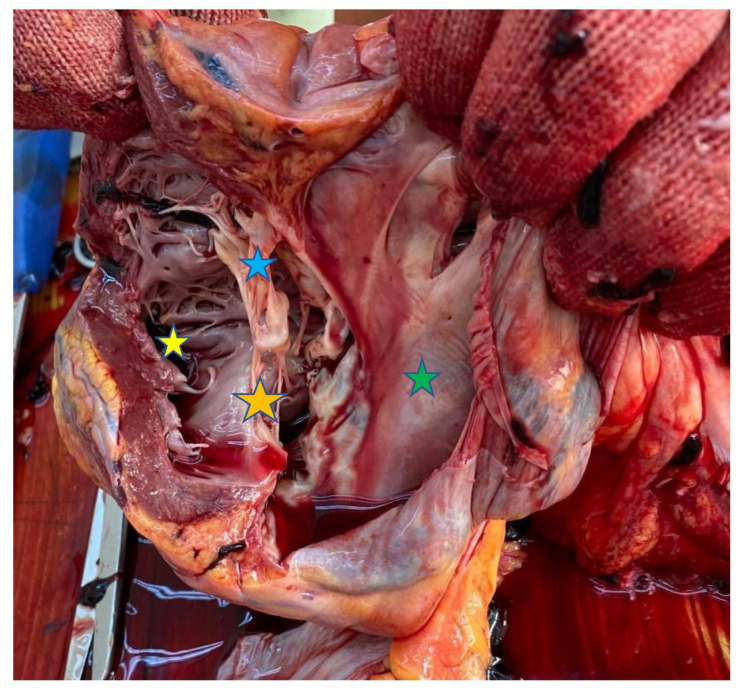
The heart was sectioned from the apex towards the base; it was globular in appearance and enlarged in volume. In one section, a purplish clot was found occupying the right heart and ascending the pulmonary arteries. The right heart was greatly hypertrophied at 1.8 cm thick. The hypoplastic left ventricle exhibited a narrowed heart chamber, and between the two atria and the two ventricles, a single valve was observed, leaving it solely responsible for the communication of all four heart chambers. An insufficiently developed interventricular septum and its congenital absence was found in the cranial third. These are the characteristic features of complete common atrioventricular canal defect with Eisenmenger syndrome [1]. The prevalence of complete atrioventricular canal is approximately 3% of all congenital heart defects, which are found in about 2 to 4 of every 10,000 live births [2]. Studies show that there is a moderately higher prevalence of congenital heart defect in females compared to males [2]. The incidence of Eisenmenger syndrome has been significantly reduced due to early diagnosis and repair of the congenital heart disease [1,2,3,4]. According to the literature, patients with Eisenmenger syndrome are not allowed to be pregnant because of high maternal and fetal risk [4]. Mothers with Eisenmenger syndrome usually die in the first three days after giving birth [4]. Yellow star: Right ventricle with a clot; right ventricular hypertrophy with a 1.8 cm myocardial wall. Green star: Left ventricle with thin walls and hypoplasticity. Blue star: Single atrioventricular valve between the four chambers. Orange star: Absence of the interventricular septum in the cranial (proximal) third.

## Data Availability

The raw data supporting the conclusions of this article will be made available by the authors on request. The data are not publicity available due to privacy reasons.
